# Building virtual patients using simulation-based inference

**DOI:** 10.3389/fsysb.2024.1444912

**Published:** 2024-09-12

**Authors:** Nathalie Paul, Venetia Karamitsou, Clemens Giegerich, Afshin Sadeghi, Moritz Lücke, Britta Wagenhuber, Alexander Kister, Markus Rehberg

**Affiliations:** ^1^ Fraunhofer Institute for Intelligent Analysis and Information Systems IAIS, Sankt Augustin, Germany; ^2^ Sanofi R&D, Translational Disease Modeling, Frankfurt, Germany; ^3^ eScience Division (S.3), Federal Institute for Materials Research and Testing, Berlin, Germany

**Keywords:** virtual patients, QSP modeling, individual patient fitting, machine learning, artificial intelligence, simulation-based inference

## Abstract

In the context of *in silico* clinical trials, mechanistic computer models for pathophysiology and pharmacology (here Quantitative Systems Pharmacology models, QSP) can greatly support the decision making for drug candidates and elucidate the (potential) response of patients to existing and novel treatments. These models are built on disease mechanisms and then parametrized using (clinical study) data. Clinical variability among patients is represented by alternative model parameterizations, called virtual patients. Despite the complexity of disease modeling itself, using individual patient data to build these virtual patients is particularly challenging given the high-dimensional, potentially sparse and noisy clinical trial data. In this work, we investigate the applicability of simulation-based inference (SBI), an advanced probabilistic machine learning approach, for virtual patient generation from individual patient data and we develop and evaluate the concept of nearest patient fits (SBI NPF), which further enhances the fitting performance. At the example of rheumatoid arthritis where prediction of treatment response is notoriously difficult, our experiments demonstrate that the SBI approaches can capture large inter-patient variability in clinical data and can compete with standard fitting methods in the field. Moreover, since SBI learns a probability distribution over the virtual patient parametrization, it naturally provides the probability for alternative parametrizations. The learned distributions allow us to generate highly probable alternative virtual patient populations for rheumatoid arthritis, which could potentially enhance the assessment of drug candidates if used for *in silico* trials.

## 1 Introduction

Quantitative Systems Pharmacology (QSP) models provide mechanistic insights into the dynamic interactions between complex pathophysiological reactions and pharmacological interventions, which yield dynamic responses of protein biomarkers and clinical endpoints (Bradshaw et al., 2019; Sorger et al., 2011). Different model parameterizations can represent variability in disease mechanisms and thereby capture a large range of patients and endotypes. An individual parameter set θ for the QSP model is here denoted as a virtual patient and determines its biomarker and disease score response to a specific treatment ( $\left.QSP\left(\theta \right)\right)$ . Finding and identifying these parameterizations θ within the disease biology network allows us to model and assess virtual patients individually and predict their disease progression and treatment response to novel drugs.

The generation of virtual patients is either driven by hypothesis, to capture, for example, high-level features of responses observed in the clinic where no data is available (Friedrich, 2016), or driven by collected clinical outcome data. Often, such data comes as summary statistics over the patient population and requires the use of parameter searches and parameter weighting methods (e.g., prevalence weighting (Klinke, 2008; Howell et al., 2012; Schmidt et al., 2013; Allen et al., 2016)). Ideally, the clinical data includes individual patient-level data which makes an explicit fit of real patients possible (Björnsson et al., 2019; Allen et al., 2021; Luo et al., 2022). The latter approach requires data preparation, high performance fitting algorithms and efficient computation pipelines to achieve a robust quantitative representation of several hundreds of patients given noisy, locally sparse and high-dimensional individual clinical data. Since the results of the individual patient fits are used to guide drug development decisions, we here seek a broad understanding of virtual patients in terms of how likely it is that they indeed describe the real patient data.

Integrating machine learning (ML) approaches to QSP modeling is a powerful strategy to tackle the computational challenges associated with mechanistic modeling of such complex biological systems (reviewed extensively in (Aghamiri et al., 2022) and (Zhang et al., 2022)). ML has been successfully implemented in parameter estimation (Wajima et al., 2009), model-order reduction (Derbalah et al., 2022), virtual patient generation (Rieger et al., 2018; Parikh et al., 2022) and the assessment of stochastic effects (McComb et al., 2022).

Here, we investigate the applicability of a novel ML approach for building virtual patients. We use simulation-based inference (SBI) that has, to the best of our knowledge, not been applied to such large QSP models yet. As an example, we use a proprietary QSP model for rheumatoid arthritis and fit it to individual patient data where patients have been treated with an anti-TNF drug. SBI approaches are advanced ML techniques for inferring a parameterization of a simulator given prior knowledge and empirical data (Lueckmann et al., 2021). While classic fitting algorithms output a point estimate for a parametrization (Byrd et al., 2000; Egea et al., 2009), SBI produces a probability distribution over the parametrization space, yielding a much more informative result. Prior knowledge in terms of an expert-designed reference patient parametrization is used to build an initial belief about the desired probability distribution. The belief then gets updated based on clinical data observations. The resulting learned probability distribution provides the probability of specific patient parameterizations and thus technically makes it possible to not only discover a single patient parameterization of high probability but multiple ones. The probability distribution could hence be used to generate new realistic virtual patients during *in silico* trials that may participate in future studies.

In a second step, we propose to leverage knowledge from already built virtual patients (from the same population) to enhance the performance of the algorithm. Instead of using the reference parametrization as prior knowledge for a new patient fit, we use an already learned parametrization of a similar patient. The so-called nearest patient fit (SBI NPF) thus starts from an improved initial belief. We expect a more consistent fit among patients of similar type, which would support an easier identification of virtual patient subgroups. To identify a similar patient, we define a vicinity criterion on the clinical data.

## 2 Methods

### 2.1 Clinical data

The individual patient data was taken from the MONARCH study [NCT02332590, anti-TNF study arm: n = 155 (Burmester et al., 2017; Gabay et al., 2017; Gaby et al., 2020)]. A total of 133 patients were used for individual patient fitting. Individual patients were fitted amongst others to cell counts (lymphocytes, macrophages), blood protein biomarkers (CRP, MMP-3, RANKL, OPG, OC, CXCL13, sICAM-1 and IL6) as well as clinical readouts (SJC28, TJC28, DAS28-CRP). The data was taken at baseline until 24 weeks of treatment with up to eight measurement time points. Population statistics of the data is available at https://zenodo.org/doi/10.5281/zenodo.12808208.

### 2.2 QSP model and simulation

The QSP model (built in SimBiology^®^, https://mathworks.com/products/simbiology.html) contains 96 ordinary differential equations (ODE) definitions, 260 reactions, 100 initial and repeated assignments and over 1,000 literature references for parameterization of 450 parameters. For simulation in Julia (version 1.8.3, https://julialang.org/), the Julia Package Sundials (package that interfaces SUNDIALS 5.2.0 library, https://github.com/SciML/Sundials.jl) with the solver CVODE_BDF() and absolute and relative tolerances of 1E-6 were used to solve the ODE system. The QSP model is shown in Supplementary Figure S1 (supplement).

The reference parametrization of the QSP model is a pre-implemented solution to an anti-TNF treatment based on various clinical, *in vitro* and animal *in vivo* experiments ranging from mechanistic to clinical outcome data (Biesemann et al., 2023).

### 2.3 Global sensitivity analysis

Global sensitivity analysis allows us to determine the importance of QSP parameters on relevant simulation outputs. The analysis was performed during drug treatment, since this is the for the parameter optimization relevant scenario. We defined the parameter ranges by a ±30% interval around the reference parametrization and used Saltelli’s sampling scheme (provided by the Python SALib module https://salib.readthedocs.io/en/latest/api.html#sobol-sensitivity-analysis, version 1.3.12). For a given parameter $\theta_{i}$ and a relevant QSP output variable $X_{j}$ we calculated the total order sensitivity index $S_{X_{j},\theta_{i}}$ following the Sobol procedure (Sobol, 2001). To deduce a single sensitivity value for each parameter $\theta_{i}$ , we aggregated the total order sensitivity $S_{X_{j},\theta_{i}}$ over the relevant output variables weighted by their variance as

$$S_{\theta_{i}}^{agg}=\frac{\sum_{j=1}^{n}S_{X_{j},\theta_{i}}Var\left[X_{j}\right]}{\sum_{j=1}^{n}Var\left[X_{j}\right]}$$ (1)

### 2.4 Simulation-based inference

Simulation-based inference (SBI) is a class of methods which apply statistical inference to learn the parameters of stochastic simulators (Lueckmann et al., 2021), and hence are applicable for learning parameters of QSP models. Statistical inference combines a prior distribution with empirical observations to conclude a posterior distribution. More precisely, given a prior probability distribution $p\left(\theta \right)$ over a parametrization $\theta \in R^{n}$ and observed data $x_{o}\in R^{d}$ , it deduces the posterior probability distribution $p\left(\left.\theta \right|x_{o}\right)$ . Following Bayes theorem (Lee, 1989), the posterior is calculated based on the likelihood function $p\left(x|\theta \right)$ . Since the analytical or numerical computation of the likelihood function is often intractable for complex simulations (Cranmer et al., 2020), SBI estimates the posterior in a “likelihood-free” manner, only relying on samples of the simulator $x\;∼\;sim\left(\theta \right)$ .

**FIGURE 1:**
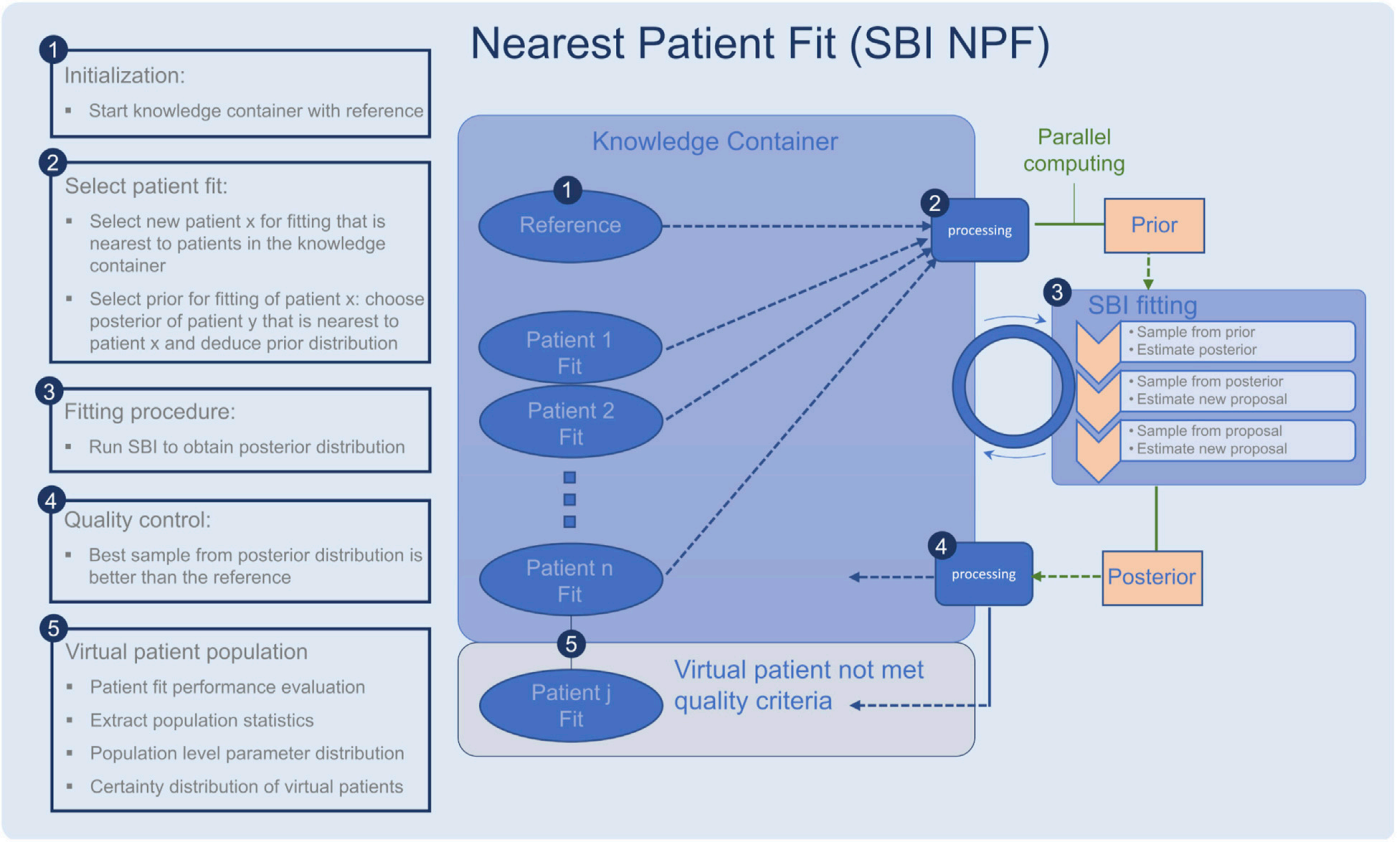
Workflow of the nearest patient fit pipeline (SBI NPF), steps 1–5. The SBI fitting procedure is depicted in step 3. Ellipses represent patient fits and boxes represent processing steps.

In this work, we evaluate an SBI approach which learns the posterior distribution with a density estimation neural network (*neural posterior estimation*). More precisely, the desired posterior $p\left(\theta \left|x\right.\right)$ is assumed to be a member of a family of probability densities $q_{\kappa }$ parametrized by κ that can be of various not-predefined shapes (e.g., multimodal). The distribution parameters κ are learned with a neural network $F\left(x,w\right)$ , where w denotes the adjustable weights of the neural network and x denotes its input, i.e., $p\left(\theta |x\right)\approx q_{F\left(x,w\right)}\left(\theta \right).$ The weights of the neural network are trained by minimizing the loss function $L\left(w\right)=\sum_{i=1}^{M}-\log q_{F\left(x_{i},w\right)}\left(\theta_{i}\right)$ over generated training samples ${\left\{\left(\theta_{i},x_{i}\right)\right\}}_{i}$ where the parameters $\theta_{i}$ are sampled from the prior $\theta_{i}\;∼\;p\left(\theta \right)$ and the corresponding simulation results $x_{i}$ are sampled from the QSP model $x_{i}\;∼\;QSP\left(\theta_{i}\right)$ . Since QSP simulations are expensive, we use the sample efficient algorithm *sequential neural posterior estimation* (Greenberg et al., 2019). Only those training samples ( $\theta_{i},x_{i}$ ) are considered relevant, where the simulation result $x_{i}$ is close to the clinical data $x_{o}$ of the patient to be fitted. Such training samples are generated by drawing parametrizations $\theta_{i}$ from a sequentially refined posterior estimate $\tilde{p}\left(\theta |x\right)$ which is called proposal posterior, *cf.* Figure 1, point 3. Since the posterior under a proposal does not coincide with the desired posterior under the prior, the authors in (Greenberg et al., 2019) present a re-parameterization of the problem to automatically transform between estimates of the proposal posterior $\tilde{p}\left(\theta |x\right)$ and the true desired posterior $p\left(\theta \left|x\right.\right)$ . The sequential procedure leads to more informative and thus overall fewer training samples from the simulator.

#### 2.4.1 Usage for individual patient fitting

To run the selected SBI approach, a variety of hyperparameters must be configured which are problem specific. First, to reduce the complexity of the optimization task, we selected an appropriate subset of the QSP parameters for fitting using global sensitivity analysis (Section 2.3) and expert knowledge. Second, we chose a prior distribution over the fitting parameters. Third, we selected the neural network-based density estimator F which models the posterior, the number of rounds in the sequential procedure of the algorithm as well as the number of samples drawn per round used to produce a posterior estimate.

Additional tuning of data and simulation outcome was applied: To handle the measurement noise of the patient data, we introduced (multiplicative lognormal) noise to the QSP simulation output during training leading to a stochastic simulator. We considered the scale parameter of the lognormally distributed noise as a fitting parameter which allowed us to regulate and learn the appropriate amount of noise per patient.

Due to the high dimensionality of the patient data, we reduced it to summary statistics for fitting. More precisely, we represented each biomarker timeseries by its median and the difference between its 0.9- and 0.1-quantile, indicating the rate with which a biomarker increases or decreases. For 16 biomarkers, this resulted in a 32-dimensional representation of the clinical data.

#### 2.4.2 Nearest patient fit (SBI NPF)

The described SBI algorithm fits each patient individually and independently. We investigated an additional approach for boosting the performance by leveraging knowledge of an already learned similar patient.

Since the raw biomarker time series data in the clinical study is sparse, we used the introduced representation of the clinical data in terms of a set of statistical features and computed the similarity between patients as the Euclidean distance in the normalized feature space. More precisely, we used the Euclidean metric proposed in (Dixon, 1979) which is designed for the presence of missing data since not all patients have measurements for all 16 biomarkers:

$$d\left(P_{i},P_{j}\right)=\sqrt{\sum_{b\in CB}\frac{2\left|CB\right|}{32}\left({\left(median\left(P_{i,b}\right)-median\left(P_{j,b}\right)\right)}^{2}+{\left(Q_{0.9}\left(P_{i,b}\right)-Q_{0.1}\left(P_{j,b}\right)\right)}^{2}\right)}$$ (2)

where CB denotes the set of common biomarkers of patient $P_{i}$ and patient $P_{j}$ , median ( $P_{i,b}$ ) is the normalized median of biomarker b for patient $P_{i}$ , and $Q_{x}\left(P_{i,b}\right)$ for $x\in \left(0,1\right)$ is the normalized x-quantile.

To implement the suggested nearest patient fit approach, we considered the fitting process of the patient cohort as a sequential procedure, *cf.* Figure 1. In each step, we fit a batch of patients in parallel and the procedure terminates when all patients are fitted. Throughout the process, the knowledge we gain from successful patient fits is collected in a so-called knowledge container which makes the knowledge available for the subsequent patient fitting experiments. The developed pipeline is described in detail in the following:

• The knowledge container is initialized with the reference patient, which is generated with the reference parametrization in the QSP model (Figure 1, step 1).
• A processing module selects a batch of patients for fitting (Figure 1, step 2), which are nearest to the current patients in the container according to our similarity metric (Equation 2). The prior for each patient fit is defined based on the learned parametrization of its most similar patient in the knowledge container.
• Each selected patient is fitted with the SBI algorithm (Figure 1, step 3).
• The quality of each resulting patient-specific posterior distribution is assessed by a processing module (Figure 1, step 4). If the learned parameterization is better than the reference parametrization according to the loss function in Section 2.6, the patient fit is put into the knowledge container. If not, its knowledge is not reused for the subsequent SBI experiments, but it is still part of our learned virtual patient population (Figure 1, step 5).

#### 2.4.3 Implementation

We chose a cross-platform implementation to combine fast and robust ordinary differential equation solvers from Julia with high performance SBI methods from Python (version 3.9.12, https://www.python.org/). We used the Python SBI implementation provided by (Lueckmann et al., 2021) and customized the simulation and patient data handling as described above. Information exchange between the SBI algorithm and the QSP model was handled using hdf5-files (in Python: https://pypi.org/project/h5py/, version 3.6.0, in Julia: https://juliaio.github.io/HDF5.jl/stable/, version v0.16.16). The fitting experiments were performed on a Linux server with Intel(R) Xeon(R) Gold 6226R 65 core CPU that has 775 GB memory available, resulting in fitting times of approximately 4 h per patient.

### 2.5 Benchmarks

Scatter search for MATLAB (SSm, Release 2014A) developed by (Egea et al., 2009) and a gradient-based method (fmincon developed by Mathworks) was used as benchmark on a Windows machine (11th Gen Intel(R) Core(TM) i7-11850H) using MATLAB R2021b and Simbiology version 6.2. Parameter bounds have been set twofold around the reference parametrization. The computation time for a single patient fit was set to 4 h, which met the convergence criterion.

### 2.6 Evaluation metrics

An individual patient fit yields a QSP parametrization θ . The quality of the parametrization was assessed by comparing the corresponding QSP output to the clinical data c as

$$L\left(\theta ,c\right)=\sqrt{\frac{1}{\sum_{b=1}^{B}T_{b}}\sum_{b=1}^{B}\sum_{t=1}^{T_{b}}{\left(\frac{{QSP}_{b,t}\left(\theta \right)-c_{b,t}}{\max\;\left(c_{b,1},\ldots ,c_{b,T_{b}}\right)}\right)}^{2}}$$ (3)

where B denotes the number of biomarkers, $T_{b}$ the number of clinical measurement time points of biomarker b , ${QSP}_{b,t}\left(\theta \right)$ the QSP output for biomarker b at time t when parametrized with θ , and $c_{b,t}$ the clinical observation of biomarker b at time t . As biomarker values may be on different scales, we used a maximum-scaling for equal weighting. Since all considered fitting algorithms (SBI, SSm, fmincon) start from the reference parametrization $\theta_{ref}$ , we evaluated their performance against the reference parameterization in terms of relative loss reduction as

$$gap\left(\theta ,c\right)=\frac{L\left(\theta_{ref},c\right)-L\left(\theta ,c\right)}{L\left(\theta_{ref},c\right)}$$ (4)

where θ denotes the parametrization determined by the respective fitting algorithm. gap <0 depicts worse data fits than the reference parameterization while gap>0 depicts improved data fits over the reference parameterization with gap=1 as the best possible case. gap=0 depicts no improvement over the reference parameterization.

For SBI, which produces a probability distribution over the parametrization, we defined the ultimate parametrization $\theta_{sbi}$ as the best one out of 100 samples drawn from the posterior. To evaluate the quality of the posterior distribution, we also reported the fraction of samples which are better than the reference parametrization,

$$frac\left(D_{post},c\right)=\frac{\sum_{k=1}^{100}1_{\left\{\left.L\right.\left(\theta_{k,sbi},c\right)<L\left(\theta_{ref},c\right)\right\}}}{100}$$ (5)

where $\theta_{k,sbi}$ denotes the *k*-th drawn sample from the learned posterior distribution $D_{post}$ and 1 is the indicator function.

## 3 Results

### 3.1 Selected hyperparameter values

**FIGURE 2:**
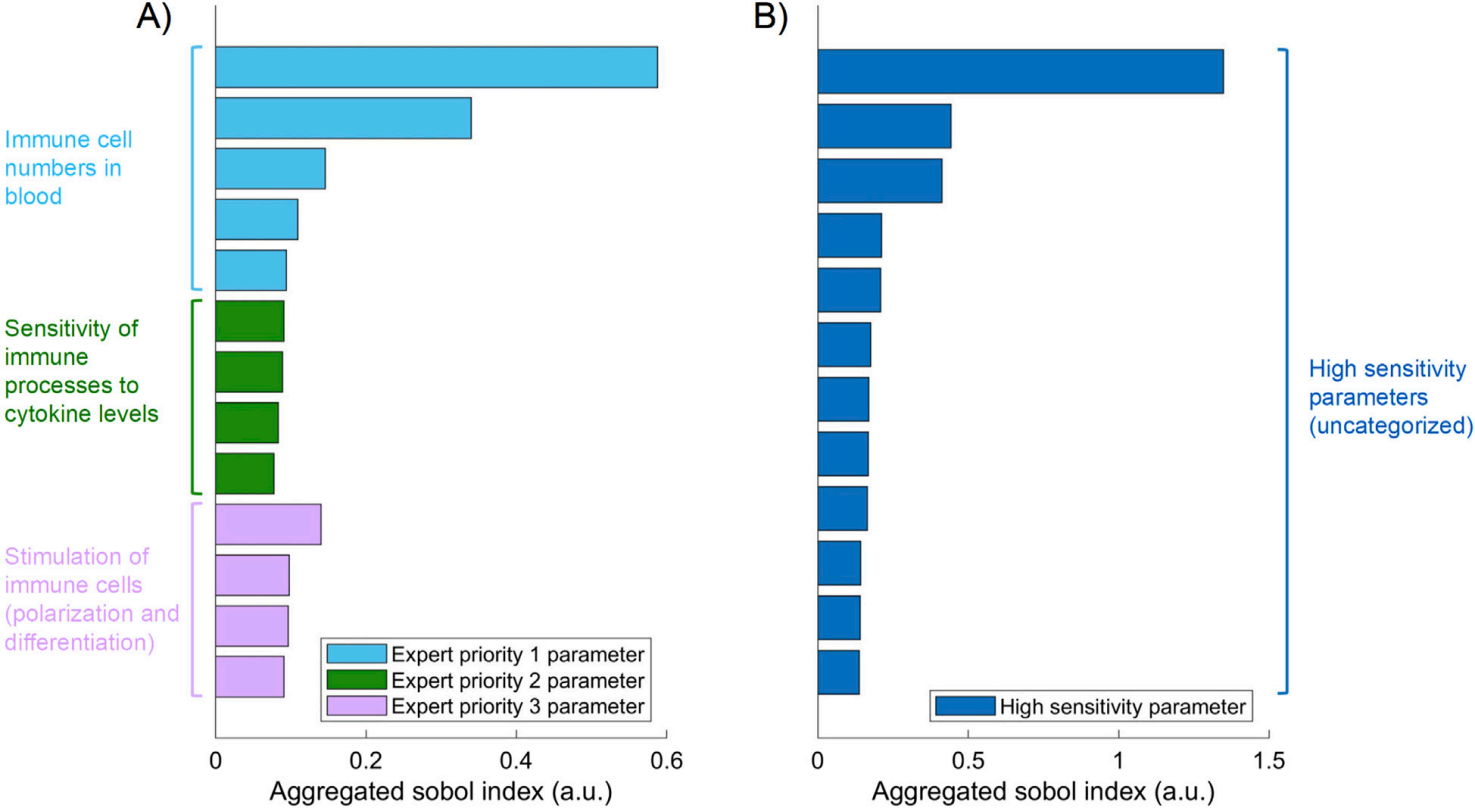
Aggregated Sobol indices for the 12 most sensitive parameters **(B)** and 13 expert priority parameters **(A)** selected by their role in the QSP RA model. Parameters are grouped by the corresponding category (color).

The hyperparameters which control the sequential training procedure (e.g., number of rounds) as well as the architecture of the density estimation neural network, were optimized with grid search (see Table 1 for an overview of the determined hyperparameter values). A relevant subset of 25 QSP parameters was selected for fitting based on biological expert knowledge, which is often a reasonable first step (Cheng et al., 2022), and global sensitivity analysis results. Figure 2 shows thirteen parameters identified as key determinants of the model output by expert priority (A), as well as the twelve most sensitive parameters (of the remaining ones) identified by global sensitivity analysis (B) (Equation 1). The parameters selected by expert priority were categorized into “immune cell numbers in blood”, “sensitivity of immune processes to cytokine levels” and “simulation of immune cells”. Variability in the expert priority parameters across virtual patients leads to variability in cell populations that play a significant role in disease pathophysiology and response to treatment. Note that the aggregated Sobol indices of the expert priority parameters are comparable to those of the high sensitivity parameters.

**TABLE 1:** Table shows results of hyperparameter tuning.

Hyperparameter	Value
Training procedure	
Number of rounds	50
Number of simulations per round	100
Prior	
Distribution	Lognormal
Prior scale	0.25
Prior loc	Reference parametrization + for noise: 0.2
Density estimator	
Neural network	“made”
Hidden features	100
Number of atoms	25

For the 25 fitting parameters we chose a lognormal prior distribution $LogNormal\left(loc,scale\right)$ centered around the reference parametrization with parameters $loc=\log\;\left(\theta_{ref}\right)$ and scale=0.25 . As the reference parametrization $\theta_{ref}$ simulates a typical patient, the prior can be an informed starting point for an individual patient fit. The lognormal distribution was chosen to keep the range of parameter values positive. Moreover, it covers the different scales of the parameters with a single *scale* value since by definition the amount of variance caused by the *scale* parameter also depends on the parameter loc (the higher loc , the higher the variance) For SBI NPF we derived the prior from the knowledge container (see Section 3.4) by centering the lognormal distribution around the parametrization of the patient’s nearest container patient (and using the same *scale* value as above).

### 3.2 Virtual patient generation

**FIGURE 3:**
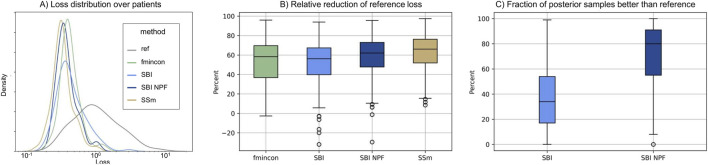
**(A)** Distribution of loss values (Equation 3) over the patient population (n = 133) for the different methods. **(B)** Distribution of the relative reduction of the reference loss over the patient population (n = 133) shown for the different methods calculated using the gap function (Equation 4). Boxes represent interquartile-ranges with a line at the median, whiskers extend to the last data point up to 1.5-fold of the interquartile range and circles represent outliers. **(C)** Distribution of the fraction of posterior samples which outperform the reference fit (ref) for both SBI approaches (SBI and SBI NPF) calculated from Equation 5.

**FIGURE 4:**
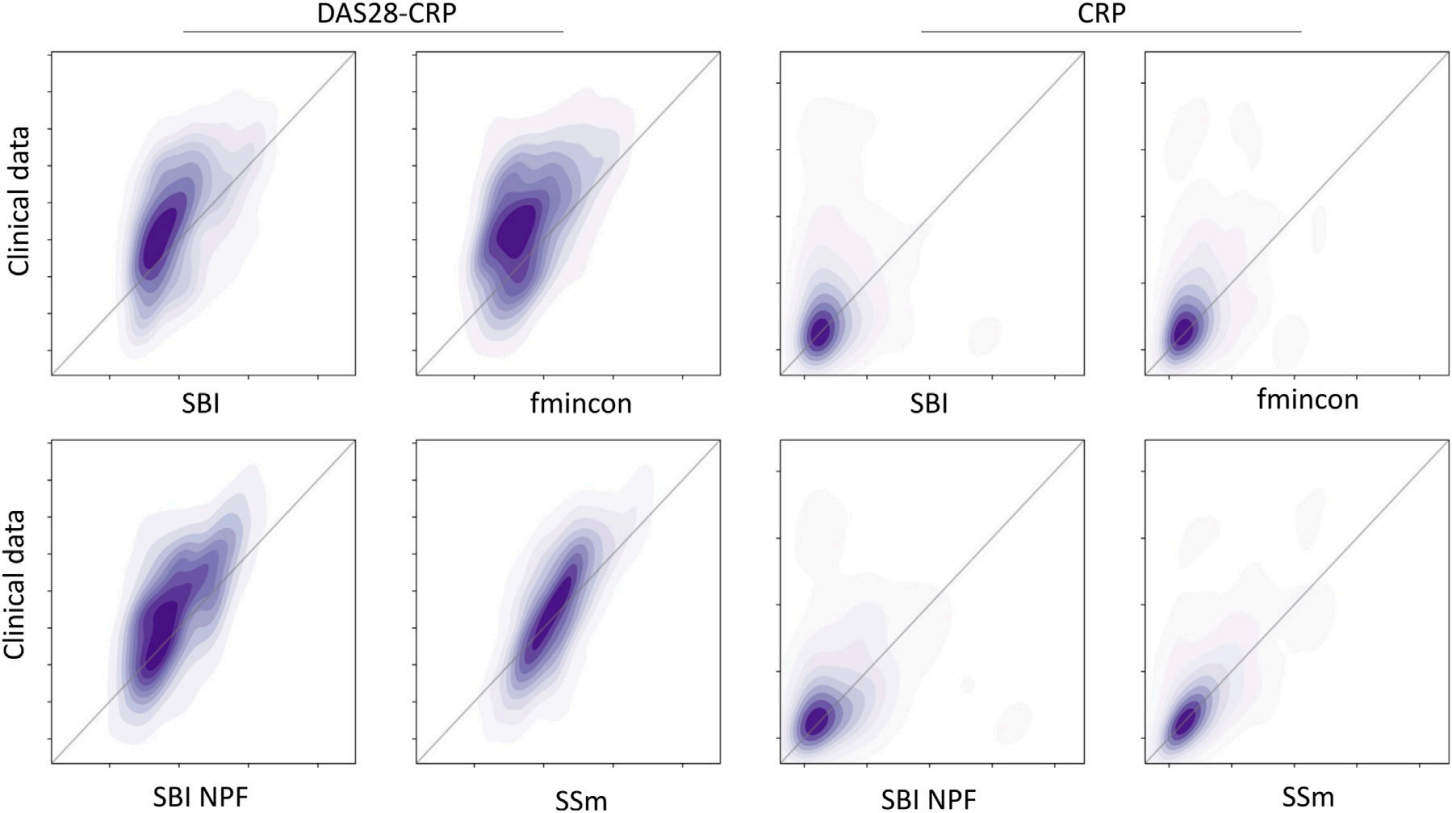
Correlation between all patient’s observations from the clinical data (*y*-axis) and the respective simulation results (*x*-axis) depicted as a density plot for a blood biomarker (CRP on the left) and a disease score (DAS28-CRP on the right). Simulation results were generated using the individual parameter estimates from the four different algorithms (SBI, fmincon, SBI NPF and SSm). Dark-shaded areas indicate high density while soft-shaded areas indicate low density.

**FIGURE 5:**
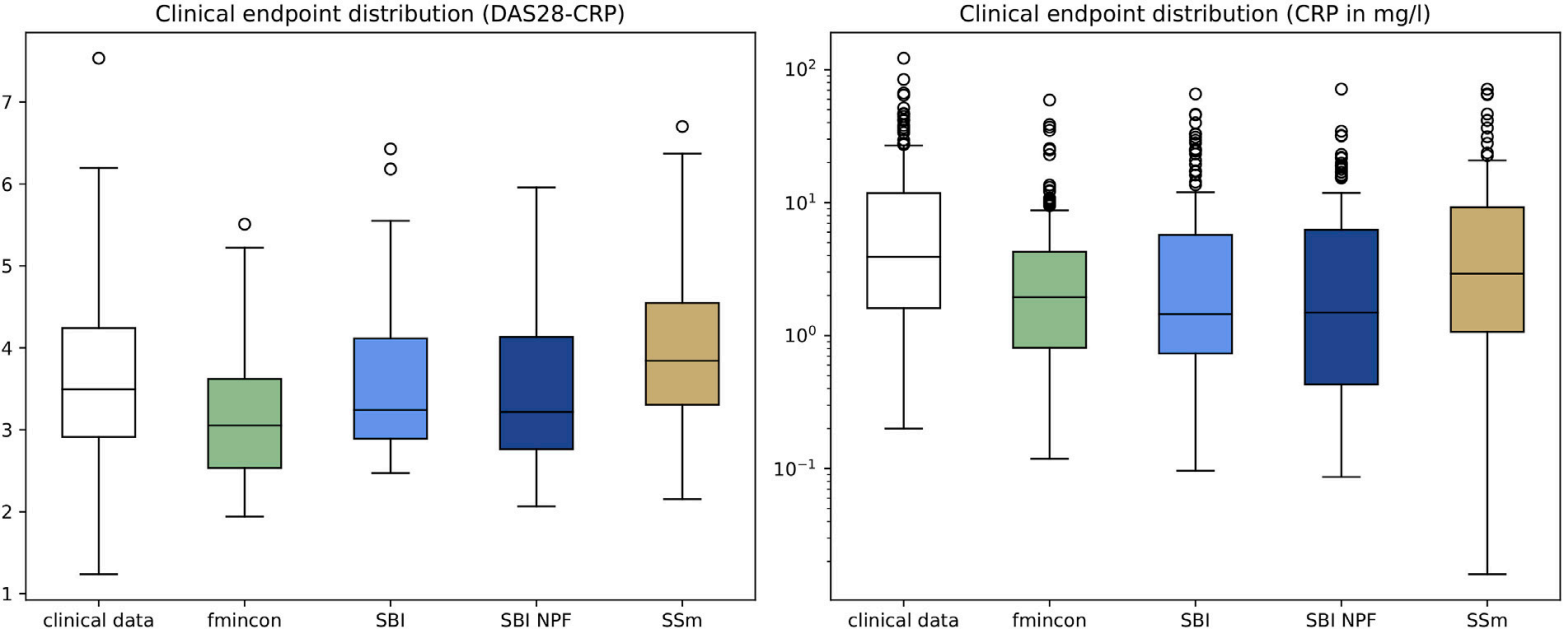
Depiction of clinical endpoints and corresponding simulation results as distributions over the patient population (n = 133) after 24 weeks of treatment for DAS28-CRP (left) and CRP (right). Simulation results were generated using the individual parameter estimates from the four different algorithms. Boxes represent interquartile-ranges with a line at the median, whiskers extend to the last data point up to 1.5-fold of the interquartile range and circles represent outliers.

Individual patient fits were performed for the presented SBI approaches as well as for the selected benchmarks (SSm, fmincon) and evaluated according to the loss function in Equation 3. For each method, the distribution of the loss over all patients is depicted in Figure 3A. All fitting algorithms lead to loss curves with smaller mean loss values and smaller variance compared to the reference. For each patient fit, the relative reduction of the reference loss is shown in Figure 3B as a distribution over the population. The fitting performance range is spanned by the two benchmarks fmincon and SSm. While the performance distribution of SBI resembles fmincon, SBI NPF yields a clear improvement which is similar to the SSm performance distribution. For both SBI approaches there are a few outlier patients, for which the reference is better than the respective SBI result (i.e., negative relative reduction in Figure 3B). When comparing the losses patient-wise, SBI NPF improves over SBI for 82% of the patients. SBI NPF does not only outperform SBI in terms of the best posterior sample but also in terms of the whole learned posterior distribution, *cf.* Figure 3C. It shows that for SBI typically 34% out of 100 posterior samples are better than the reference parametrization, while for SBI NPF this number is around 80%. Visual predictive checks on a biomarker level are presented in Figures 4, 5 for the c-reactive protein(CRP) and a disease score (DAS28-CRP). Figure 4 compares the clinical biomarker observations of all patients (*y*-axis) at all time points to the corresponding simulations of the model with the parameter sets of the respective fitting algorithm (*x*-axis). This density correlation plot illustrates that, similar to the benchmarks, the SBI approaches are overall able to describe the clinical data sufficiently well. The visual predictive checks also reflect that SBI NPF leads to better fits than SBI. In Figure 5 we depict the distribution of the clinical data and the obtained simulation results (after 24 weeks of treatment) over the patient population. Inter-patient variability is large in the clinical data endpoints and the fitting methods are generally able to capture this variability under the chosen parameter bounds. An example of an individual fit obtained by SBI is shown in the Supplementary Figure S2 for the CRP data. In summary, the empirical evaluations demonstrate that the SBI approaches can compete with classic fitting methods in the field in terms of fitting quality and fitting speed. Moreover, the suggested SBI NPF pipeline significantly improved over SBI.

### 3.3 Comparison of virtual patients

**FIGURE 6:**
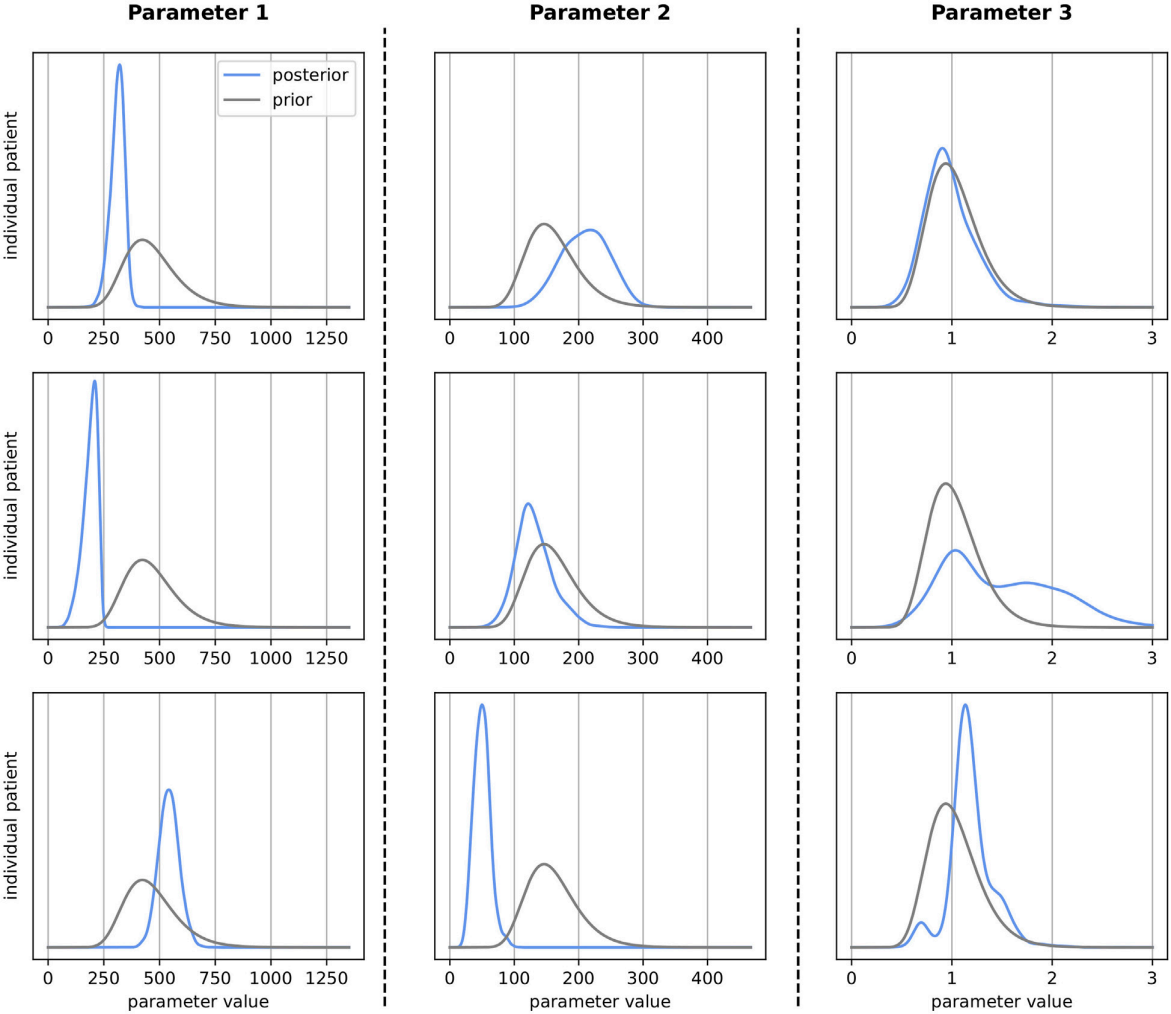
Prior (grey) vs. selected patient-individual posterior (blue) one-dimensional marginal distributions for three model parameters. Every subplot stands for an individual patient. Each column represents one specific parameter. *X*-axes represent the parameter value used in the QSP model (parameter-specific units) and *y*-axes represent the density.

For each patient, SBI produces a posterior probability distribution over the considered 25-dimensional parameter space. Exemplary one-dimensional marginal posteriors are depicted in Figure 6 for three different parameters. One column depicts the marginal distribution for a specific QSP model parameter for three different patients which all started from the same prior (grey). For each parameter (column), the three learned patient-individual posteriors (blue) differ significantly from each other. While a learned posterior can have moved far away from the prior, i.e., the reference parametrization, they can also resemble each other, at least in the one dimension depicted in this figure (similarity of the here depicted one-dimensional marginal prior and posterior does not imply similarity of the 25-dimensional prior and posterior distributions). Overall, we observe multiple shapes of the marginal posteriors, which range from very concentrated distributions to broader and flat ones.

Note that the sampled parameter sets from a patient posterior distribution contain between-parameter relationships (example given in the supplement as parallel coordinate plot, see Supplementary Figure S3) and can be used to explore correlations (example given in the supplement as correlation matrix, see Supplementary Figure S4).

## 4 Discussion

### 4.1 Concept: Generation of virtual patients by fitting individual patient data

QSP models are typically built in several steps. Individual mechanistic parameters, such as binding or dissociation as well as mechanistic pathway modules are first calibrated based on *in vitro* and *in vivo* experiments and, in the final step, are then fitted to clinical study data such as biomarker concentrations and disease activity endpoints (Cheng et al., 2022).

Often this clinical data is only available as summary statistics, which requires weighting methods to ensure a proper distribution of the inferred parameter sets (Klinke, 2008; Schmidt et al., 2013). It requires difficult assumptions on which patients may exist in the real world and has consequences for prediction of drug efficacy.

Fitting of individual clinical data circumvents these assumptions but is limited, in good cases, to only a few hundred patients where the individual data is often provided without uncertainty statistics (such as standard deviation). The lack of uncertainty statistics denies the use of sophisticated approaches for generating alternative parameterizations for a single patient, such as bootstrapping (Tibshirani and Efron, 1986).

By applying a simulation-based inference method, we generated parameter probability distributions during the patient fit, directly providing alternative parametrizations for real patients. More precisely, sampling from the probability distributions yield different highly likely parametrizations for an individual patient, which can then be used to achieve a larger virtual patient population. Thus, the above-mentioned limitations of individual patient data have been overcome and the generated virtual population is based on real patients, which is advantageous compared to weighting methods and their assumptions.

The subsequent validation of the generated virtual population, either from individual patient fitting or from hypothesis-based methods, is usually achieved by predicting the population outcome of other studies, for example, drugs with different mode of action or different dosing schemes, under consideration of the baseline characteristics of the study population.

### 4.2 SBI for fitting individual patient data

In this work, we employ SBI to learn a distribution over the QSP model parametrization for an individual rheumatoid arthritis patient and build a virtual patient from it. The goal is to identify regions in the parameter space which best explain the patient observations, i.e., where the corresponding simulated biomarker values match the patient’s clinical data.

The approach is particularly interesting for the described setup since there may exist multiple optimal QSP parametrizations to model the patient data. The learned probability distribution in the parameter space naturally provides the probability of certain parameterizations and can be used to explore alternative parameterizations. Another benefit of SBI is that it treats the simulation as a black box, similar to SSm and fmincon.

There exists a variety of SBI algorithms in the literature, see (Lueckmann et al., 2021) for a detailed overview, from which we chose the sample-efficient algorithm *sequential neural posterior estimation.* The choice of the SBI approach as well as of the stochastic global and deterministic local approach can yield differences in the benchmarking as their performance needs to be considered as partially problem specific (Egea et al., 2009). Furthermore, the applied data statistics and the data noise handling can influence the result performance.

### 4.3 Choice of hyperparameters

Within this challenging optimization problem, algorithms and settings of hyperparameters are an impactful choice that is based on the underlying optimization criterion and performance assumptions. Alternative hyperparameter settings may yield similar or better results and can be subject of further analysis.

To reduce the complexity of the optimization problem and to achieve high quality model fits, we selected the most relevant parameters for model fitting by assessing the parameter influence on biomarker-related model outputs through global sensitivity analysis (Sobol, 2001). In addition, expert priority parameters have been included in the parameter estimation (Cheng et al., 2022). The quantitative choice of 25 parameters seems arbitrary but alternative parameter numbers did not improve the result of the parameter estimation.

### 4.4 Performance of virtual patient generation

The results of this work demonstrate that fitting of individual patients can yield virtual patients that each outperform the reference and that the model parameterizations can represent the variability in clinical response typically seen in the data. The variability in the patient data was very high, *cf.* Figure 5, which is expected for rheumatoid arthritis as heterogeneous disease, and poses a real challenge for individual patient fitting but also for predicting response (Rehberg et al., 2021). Obviously, the inter-patient variability is a consequence of phenotypic differences and measurement noise. As noise cannot be explained biologically with the mechanistic QSP model, a perfect correlation between clinical data and model predictions in Figure 4 is difficult to achieve (see also (Schmidt et al., 2013)). Yet the discussed algorithms show a different fitting performance with fmincon performing worst, SSm performing best and SBI being in between. Fmincon generally is less suited for our optimization task than the others as it searches for a local and not necessarily global optimum. While fmincon and SSm provide only point estimates, SBI provides a distribution, i.e., multiple parameter estimates with corresponding probabilities. We note that the fitting approach with SBI uses summary statistics of the clinical data and not its raw observations like the benchmarks, which could be a disadvantage. Yet overall, the SBI approaches get reasonably close to SSm. Our results also illustrate that SBI can handle a high-dimensional parameter space of 25 parameters and make them suited for such kind of QSP problems. For comparison, SBI approaches in the literature focused, so far, on setups of only 2–10 parameters (Lueckmann et al., 2021; Reza et al., 2022; Boelts et al., 2023; Boyali et al., 2021). The fact that SBI could be improved with SBI NPF for 82% of the patients demonstrates a high potential of the nearest patient fit pipeline developed in this work. It showcases the influence and necessity of good prior estimates for SBI algorithms. However, 18% of the patients were better fitted with SBI, which starts from a presumably less appropriate prior distribution. While SBI approaches are inherently stochastic, the impact on fitting quality was minor in repetitive experiments. We must assume that the SBI NPF pipeline has room for improvement in defining the patient vicinity criteria and/or that patient vicinity is not always of benefit, as a QSP model may require very different parametrizations to produce similar outputs (Duffull and Gulati, 2020). To conclude on the SBI NPF pipeline, the developed concept of nearest patient fits is not specific to SBI but represents a generic contribution that can be transferred to any fitting algorithm which considers initial solutions.

### 4.5 Comparison of virtual patients

The patient-specific posterior marginal distributions show that very diverse QSP model parametrizations can be necessary to describe individual patients well, which SBI was able to learn. The different shapes of the marginal posteriors indicate the flexibility of the chosen SBI approach (sequential neural posterior estimation) in modelling probability distributions. While concentrated distributions can indicate a high certainty in the virtual patient parametrizations, flat distributions may point towards those that are uncertain. One advantage of the learned distributions is that alternative virtual patient parametrizations can directly be generated through sampling. I.e., new highly-probable patient fits can be easily generated without re-running the optimization solver or using other metrics and assumptions such as prevalence weighting. These alternate parameterizations of a virtual patient may describe the fitted data equally well and may represent differences in the disease mechanisms. Exploring alternate parametrizations is fundamental to assess the range of treatment outcomes of an individual patient.

On the population level, aggregation of the given patient-specific posterior distributions may allow the application of population statistics for assessment of subgroups, patient differences and population spread.

### 4.6 General conclusion

In this work, we find SBI approaches to be powerful tools in creating virtual patients using individual patient data. SBI achieved the same performance in patient fits compared to benchmark algorithms and provides parameter probability distributions, which can be used to explore alternative parameterizations for real patients to create more confidence in predicting clinical outcomes for *in silico* trials. Furthermore, leveraging patient similarities observed in the clinical data, improved the performance and may be suited as a generalizable strategy in generating virtual patients.

## Data Availability

The original contributions presented in the study are included in the Supplementary Material, further inquiries can be directed to the corresponding author.

## Supplementary material

SUPPLEMENTARY FIGURE S1 Schematic representation of key interactions in the QSP RA model. The model is composed of a blood compartment (from which immune cells are recruited) and a synovial tissue compartment (side of inflammation). Thickness of connection dots illustrate the influence strength. Dots represent positive influence, bars represent negative influence (only for Tregs). Abbreviations: TNF: tumor necrosis factor, FLS: fibroblast-like synoviocytes IL6R: interleukin-6 receptor, CRP: c-reactive protein, DAS28-CRP: disease activity score 28 with CRP, TH: T helper cells, MAC: macrophages, Treg: T regulatory cells, B: B cells.

SUPPLEMENTARY FIGURE S2 Individual patient fit of QSP model to c-reactive protein (CRP) data obtained by SBI. The clinical patient data is depicted as circles. Data before treatment start shows baseline characteristics of that individual patient and the drop in CRP shows response to the treatment (treatment time: 24 weeks). Each dashed blue line represents a fit obtained by SBI. More precisely, it represents the QSP simulation result when parametrized with a sample from the learned patient posterior. Note that the depicted fitting result was obtained by fitting 16 clinical biomarkers and endpoints from that patient simultaneously (including CRP).

SUPPLEMENTARY FIGURE S3 Parallel coordinate plot of parameter sets sampled from a patient posterior distribution obtained by SBI. The 25 fitting parameters are depicted as p1, …, p25 as columns, each equipped with a *y*-axis showing the respective parameter value range. Each line (from p1 to p25) is one parameter set derived from SBI for the given patient, obtained by sampling from the learned patient posterior distribution. A line’s color represents the quality of that parameter set in terms of the resulting fitting loss (Equation 3). Parameter sets in dark green color illustrate low loss values while orange parameter sets have higher loss values. A total of 100 parameter sets is shown.

SUPPLEMENTARY FIGURE S4 Parameter correlation calculated for the parameter sets shown in Supplementary Figure S3: The heatmap shows the correlation between the 25 parameters shown as p1, …, p25 obtained from the 100 parameter sets depicted in Supplementary Figure S3. Numbers are Pearson correlation coefficients and are highlighted in red for positive correlation and in blue for negative correlation.
