# Supplementary figures and images for: Building virtual patients using simulation-based inference
Source: Front Syst Biol. 2024 Sep 12;4:1444912. doi: 10.3389/fsysb.2024.1444912 (PMC12342008; doi:10.3389/fsysb.2024.1444912)

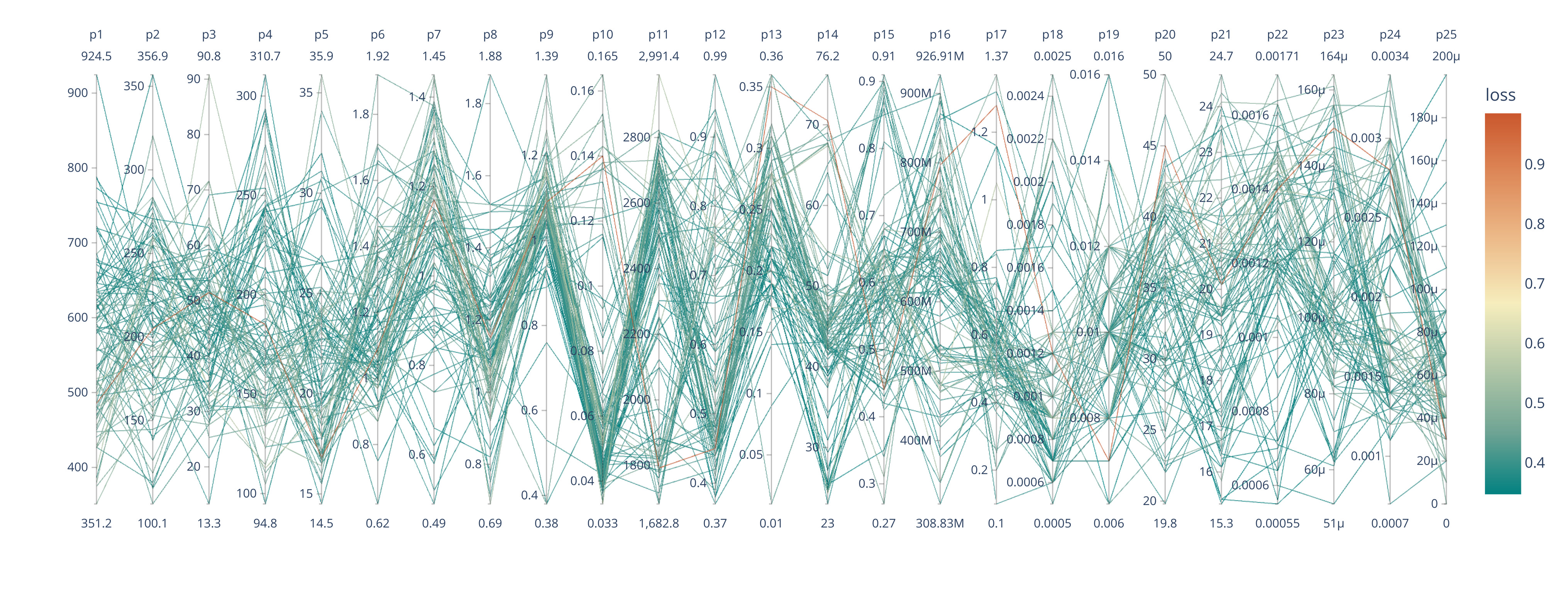

Supplement: Supplementary file 1 [file Image3.JPEG]

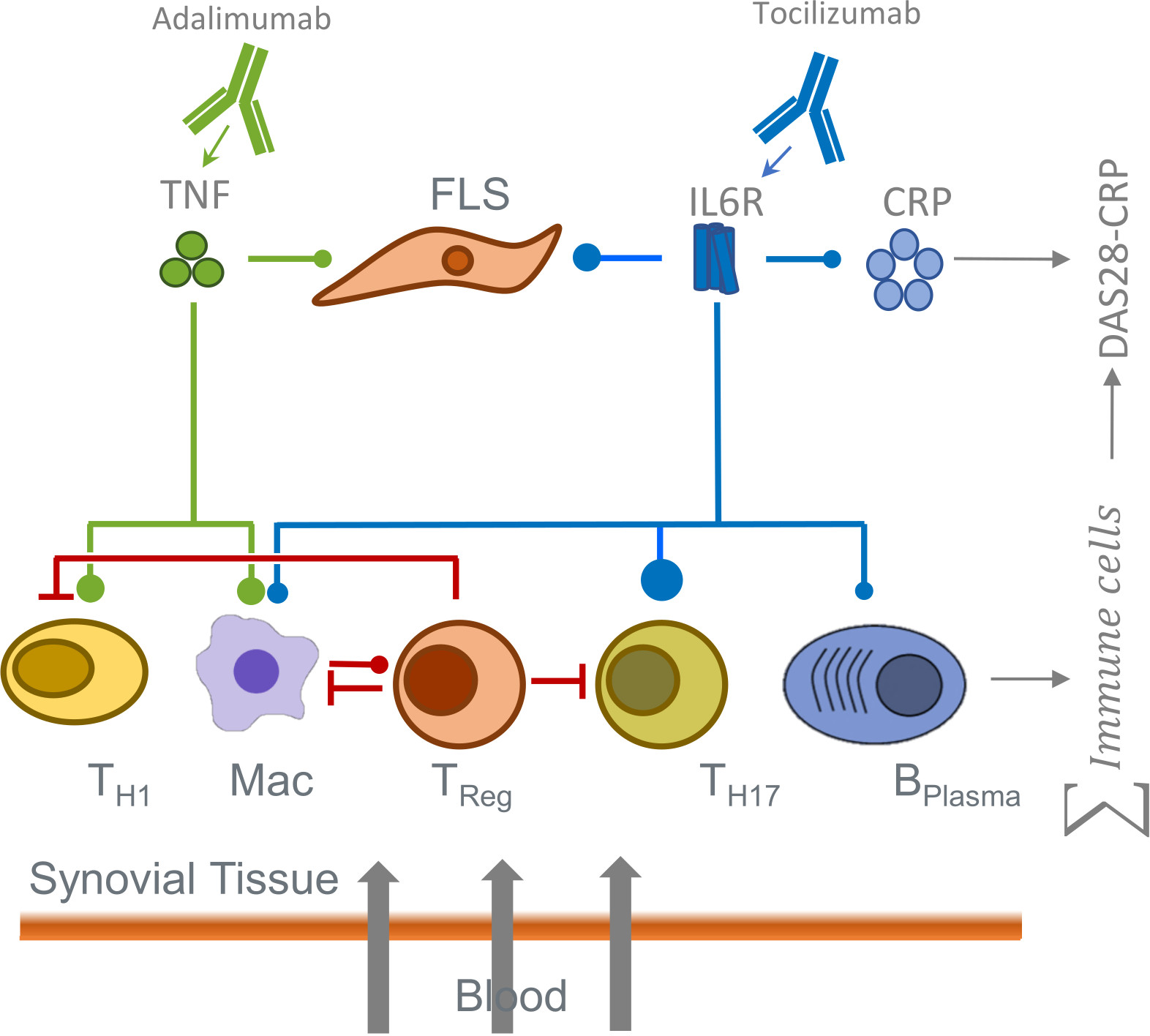

Supplement: Supplementary file 2 [file Image1.JPEG]

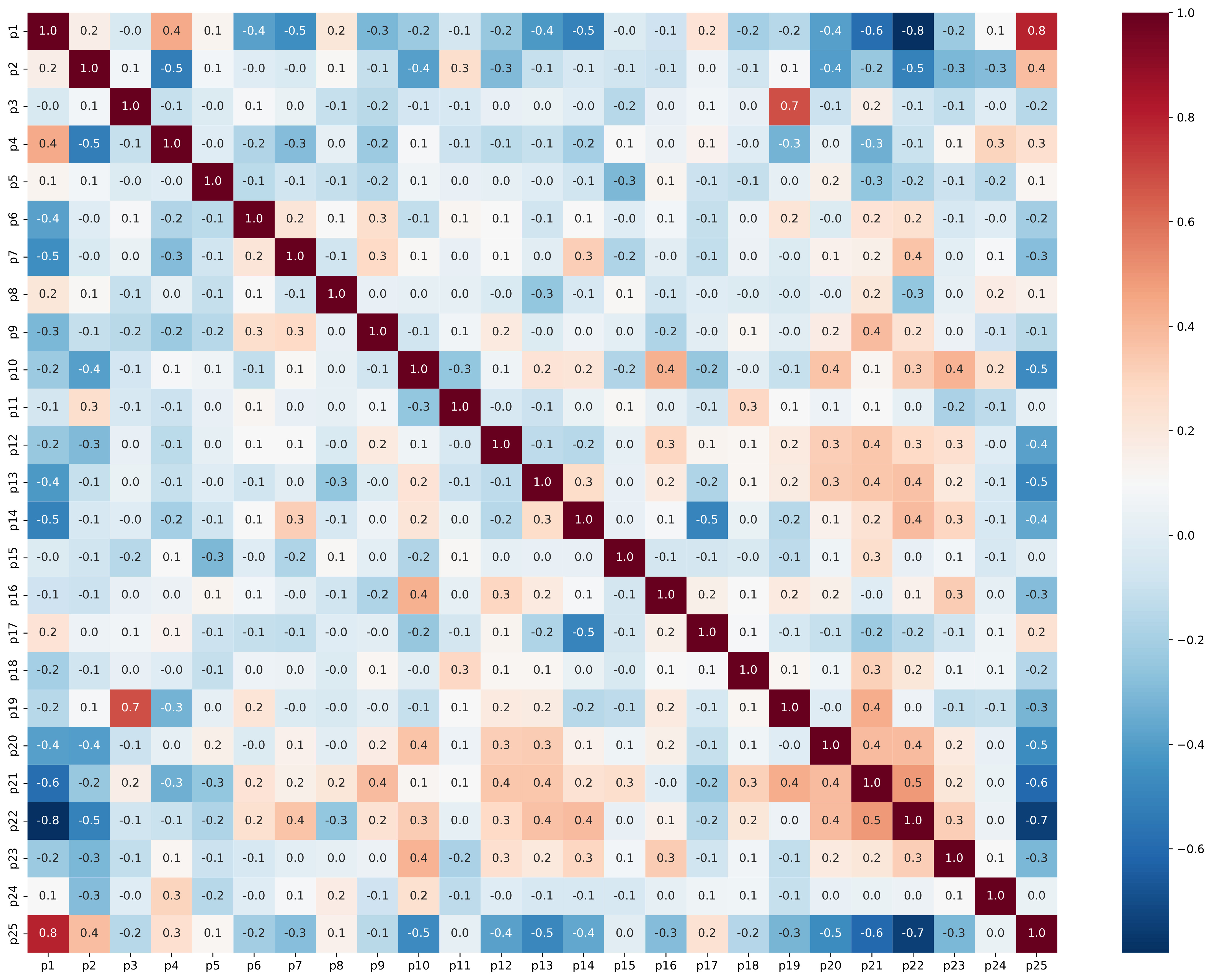

Supplement: Supplementary file 3 [file Image4.JPEG]

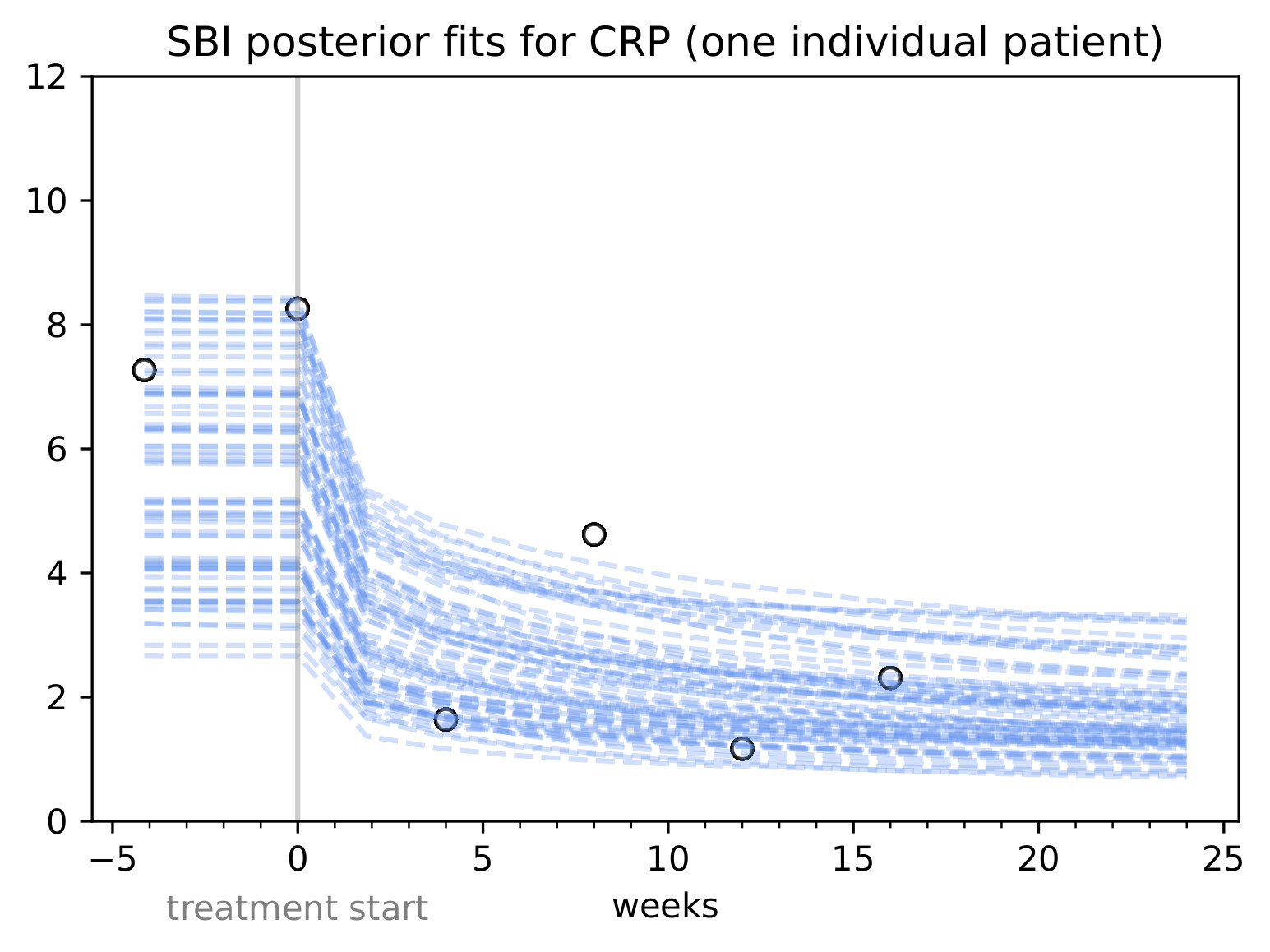

Supplement: Supplementary file 4 [file Image2.JPEG]
