# Supplementary material for: Building virtual patients using simulation-based inference
Source: Front Syst Biol. 2024 Sep 12;4:1444912. doi: 10.3389/fsysb.2024.1444912 (PMC12342008; doi:10.3389/fsysb.2024.1444912)
Supplement: Supplementary file 5 [file DataSheet1.DOCX]

# Supplement

**Supplementary Figure S1): Schematic representation of key interactions in the QSP RA model.** The model is composed of a blood compartment (from which immune cells are recruited) and a synovial tissue compartment (side of inflammation). Thickness of connection dots illustrate the influence strength. Dots represent positive influence, bars represent negative influence (only for Tregs). Abbreviations: TNF: tumor necrosis factor, FLS: fibroblast-like synoviocytes IL6R: interleukin-6 receptor, CRP: c-reactive protein, DAS28-CRP: disease activity score 28 with CRP, TH: T helper cells, MAC: macrophages, Treg: T regulatory cells, B: B cells.

**Supplementary Figure S2): Individual patient fit of QSP model to c-reactive protein (CRP) data obtained by SBI.** The clinical patient data is depicted as circles. Data before treatment start shows baseline characteristics of that individual patient and the drop in CRP shows response to the treatment (treatment time: 24 weeks). Each dashed blue line represents a fit obtained by SBI. More precisely, it represents the QSP simulation result when parametrized with a sample from the learned patient posterior. Note that the depicted fitting result was obtained by fitting 16 clinical biomarkers and endpoints from that patient simultaneously (including CRP).

**Supplementary Figure S3): Parallel coordinate plot of parameter sets sampled from a patient posterior distribution obtained by SBI.** The 25 fitting parameters are depicted as p1, …, p25 as columns, each equipped with a y-axis showing the respective parameter value range. Each line (from p1 to p25) is one parameter set derived from SBI for the given patient, obtained by sampling from the learned patient posterior distribution. A line’s color represents the quality of that parameter set in terms of the resulting fitting loss (Eq. 3). Parameter sets in dark green color illustrate low loss values while orange parameter sets have higher loss values. A total of 100 parameter sets is shown.

**Supplementary Figure S4): Parameter correlation calculated for the parameter sets shown in supplementary Figure S3:** The heatmap shows the correlation between the 25 parameters shown as p1, …, p25 obtained from the 100 parameter sets depicted in Figure S3. Numbers are Pearson correlation coefficients and are highlighted in red for positive correlation and in blue for negative correlation.
